# Efficacious Anti-Cancer Drugs Targeting Nicotinamide *N*-Methyltransferase (NNMT) in Cultured Human Oral Squamous Cell Carcinoma (OSCC)

**DOI:** 10.3390/ph19030516

**Published:** 2026-03-22

**Authors:** Brian Maloney, Martyna Kubisztal, Ziqian Ge, Yin Lu, Lisa Strotmann, Adrianna Budziňska, Mary F. Rooney, Marilena Karavyraki, Andrew Knox, Richard K. Porter

**Affiliations:** 1 School of Dental Science, Trinity College Dublin, D02 F859 Dublin, Ireland; maloneb3@tcd.ie; 2 School of Biological and Health Sciences, Technical University of Dublin, D07 XT95 Dublin, Ireland; martinakub13@gmail.com (M.K.); andrew.knox@tudublin.ie (A.K.); 3 School of Biochemistry & Immunology, Trinity College Dublin, D02 R590 Dublin, Ireland; gezi@tcd.ie (Z.G.); lu.yin@tudublin.ie (Y.L.); marooney@tcd.ie (M.F.R.); karavyrm@tcd.ie (M.K.); 4 Institute of Biochemistry, Albert-Ludwigs University Freiburg, Albertstraβe 21, 79104 Freiburg im Breisgau, Germany; 5 Laboratory of Mitochondrial Biochemistry, Adam Mickiewicz University, 61-714 Poznań, Poland; adrianna.budzinska@amu.edu.pl

**Keywords:** oral squamous cell carcinoma, nicotinamide *N*-methyltransferase, mitochondria, cellular oxygen consumption, SCC-4, DOK, MCF 7, AG-670, AO-022

## Abstract

**Background/Objectives:** Oral squamous cell carcinoma (OSCC) is a major cause of human cancer. The enzyme, nicotinamide *N*-methyltransferase (NNMT), is overexpressed in a variety of human cancers, including OSCC. Our objective was to target NNMT with novel inhibitors and determine their anti-cancer efficacy while shedding light on their possible mechanism of action. **Methods:** We identified two small molecule inhibitors of NNMT (AG-670 and AO-022) based on a pharmacophore of the in silico nicotinamide binding site. These inhibitors were investigated for (i) potency to inhibit the activity of the isolated NNMT enzyme (EC_50_ values), (ii) cytotoxicity (IC_50_ values) against the human OSCC cell line, SCC-4, and (iii) ability to affect cellular energy metabolism, as measured by oxygen consumption, in SCC-4 cells (plus dysplastic oral keratinocytes (DOK) cells and breast cancer MCF-7 cells). Immunoblotting was used to determine whether NNMT was expressed in the aforementioned cells. **Results:** NNMT is expressed in SCC-4 and DOK cells (and primary human oral keratinocytes) but not MCF 7 cells. The NNMT inhibitors inhibit isolated NNMT enzyme activity and were cytotoxic to SCC-4 cells (EC_50_ and IC_50_ values in the micromolar range). Sublethal doses of the inhibitors were demonstrated to inhibit in situ mitochondrial oxygen consumption in SCC-4 and DOK cells but not in MCF-7 cells. It was demonstrated that the NNMT inhibitors do not directly inhibit mitochondrial electron transport chain activity. Thus, we deduce that the NNMT inhibitors affect mitochondrial activity indirectly via NNMT. **Conclusions:** It is concluded that NNMT is a potential drug target for oral cancer.

## 1. Introduction

**Figure 1:**
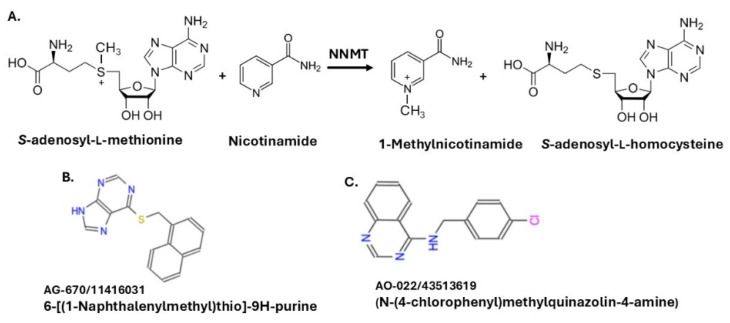
Depiction of the enzyme activity catalyzed by nicotinamide *N*-methyltransferase (NNMT) and representative structures of the NNMT inhibitors (AG-670 and AO-022) investigated. (**A**) Nicotinamide *N*-methyltransferase (NNMT) is a one-carbon group cytosolic enzyme that is involved in the catalysis of methylation, by S-adenosyl-L-methionine, of nicotinamide thus generating S-adenosyl-L-homocysteine and 1-methylnictotinamide [12,13]. The two small molecule inhibitors of NNMT (**B**) AG-670/11416031 (AG-670) and (**C**) AO-022/43513619 (AO-022) were selected based on in silico analysis defining the pharmacophore of the nicotinamide binding site of NNMT (Supplementary Section S1).

Oral squamous cell carcinoma (OSCC) accounts for up to 90% of all oral malignancies and is the sixth most common cancer worldwide [1]. Treatment involves surgical removal of the tumor, radiation, and chemotherapy although such treatments are not always satisfactory with 5-year survival of only 50% being recorded [1,2]. We were interested in a more targeted approach to compliment the established cancer therapeutics. The enzyme, *N*-nicotinamide methyltransferase (NNMT), is overexpressed in a variety of human cancers, including oral squamous cancer biopsies [3], and has recently been investigated by several laboratories as a potential anti-cancer target [4,5,6,7,8,9,10,11]. Nicotinamide *N*-methyltransferase (NNMT) catalyzes the methylation of nicotinamide (NAM) by transferring the methyl group the methyl donor S-adenosyl-L-methionine to nicotinamide, subsequently generating S-adenosyl-L-homocysteine and 1-methylnictotinamide [12,13] (Figure 1A). NNMT thus modulates intracellular NAD^+^ levels, which are crucial for energy metabolism, oxidative phosphorylation [14,15] and metabolic enzyme activity via NAD^+^-dependent sirtuin deacetylases [16].

Under normal physiologic conditions, NNMT is predominantly expressed by hepatocytes in the liver, white adipose tissue, skeletal muscle and lung, while multiple mesenchymal cell types have lower levels of NNMT expression [17,18]. However, numerous studies have suggested that NNMT expression is significantly increased in several kinds of cancer, including neuroblastoma [19], oral squamous cell carcinoma [3], papillary thyroid cancer [20], lung cancer [21], breast cancer [22], gastric cancer [23], pancreatic cancer [24], colorectal cancer [25], renal carcinoma [26], and ovarian clear cell carcinoma [27]. High NNMT expression in these cancers appears to be inversely associated with the tumor size and progression, suggesting that NNMT may have potential effects in an initial step of malignant conversion [28]. However, and interestingly, there are also cancer cell lines where NNMT is not expressed such as in the estrogen-receptor positive breast cancer cell line MCF 7 [29,30].

Several NNMT inhibitors have been identified to date, such as methylated quinolines [6], nicotinamide analogs [7,8], covalent inhibitors [4,9], and amino-adenosine and alkynyl derived bi-substrate inhibitors [5,10,11]. Our laboratory identified two small molecule inhibitors of NNMT (AG-670 and AO-022) (Figure 1B,C) based on a pharmacophore of the in silico nicotinamide binding site (Supplementary Section S1). We know from the aforementioned previous studies that NNMT is an anti-cancer target in many tissues. We hypothesized that the inhibition of NNMT would be cytotoxic to OSCC and, through the very nature of the enzyme impacting NAD^+^-levels, have a downstream effect on oxidative phosphorylation. The effect of these modulators (AG-670 and AO-022) of NNMT activity on OSCC cell viability and energy metabolism was evaluated.

## 2. Results

**Figure 2:**
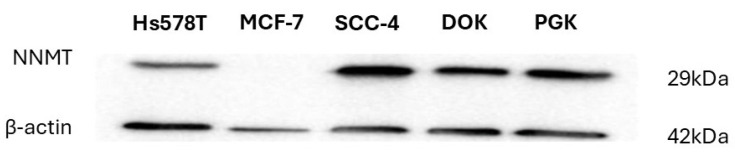
Immunoblot of nicotinamide *N*-methyltransferase (NNMT) expression in various cultured cells. NNMT is expressed in Hs578T cells (a triple negative breast cancer cell line), SCC-4 cells (an oral squamous cell carcinoma cell line), DOK cells (a pre-cancerous dysplastic oral keratinocytes cell line) and primary oral gingival keratinocytes (PGK cells). NNMT is not expressed in the estrogen (ER), progesterone (PR), and glucocorticoid receptor-positive MCF7 cells.

The enzyme, nicotinamide *N*-methyltransferase (NNMT), is overexpressed in a variety of human cancers. Figure 2 demonstrates that NNMT is expressed in Hs578T cells (a triple negative breast cancer cell line), SCC-4 cells (an oral squamous cell carcinoma cell line), DOK cells (a pre-cancerous dysplastic oral keratinocytes cell line) and primary oral gingival keratinocytes (PGK cells). However, Figure 2 demonstrates that NNMT is not expressed in estrogen (ER), progesterone (PR), and glucocorticoid receptor-positive MCF7 cells, confirming data presented by others [29,30].

**Figure 3:**
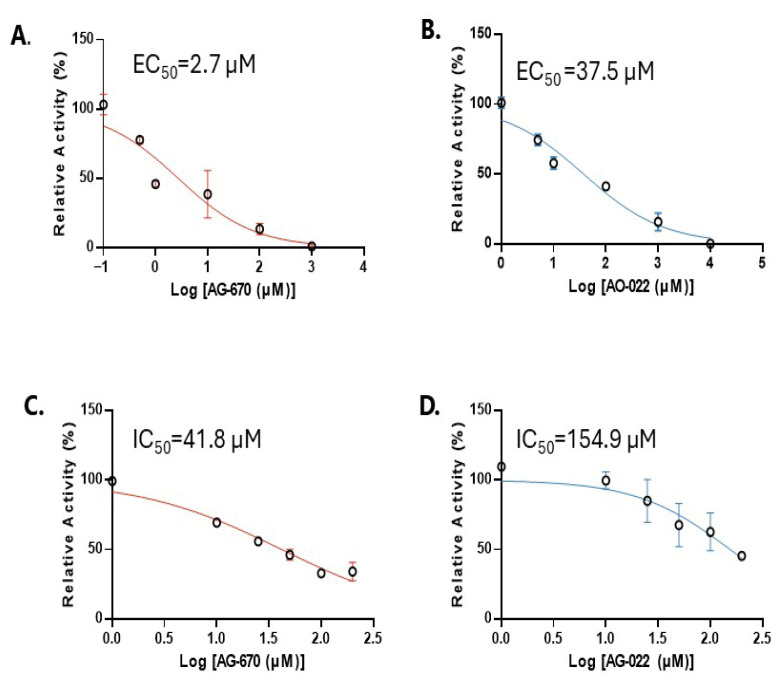
The effect of AG-670 and AO-022 on nicotinamide *N*-methyltransferase (NNMT) isolated enzyme activity (EC_50_) and SCC-4 cell cytotoxicity (IC_50_). (**A**,**B**) An NNMT inhibitor screening assay kit (Sigma-Aldrich) was used to determine the potency of potential NNMT inhibitors (AG-670 and AO-022) on the isolated enzyme (EC_50_). (**C**,**D**) SCC-4 cell viability was determined by Alamar blue assay using a range of NNMT inhibitor concentrations over 72 h using 1% FBS (IC_50_).

Figure 3 demonstrates the potency of the two small molecule inhibitors (AG-670 and AO-022) on isolated NNMT enzyme activity and SSC4 cell cytotoxicity. Figure 3A,B demonstrate that AG-670 and AO-022 directly inhibit enzyme activity with EC_50_ values of 2.7 µM (95% CI: 1.4–5.8) and 37.5 µM (95% CI: 23.4–60.5), respectively. Figure 3C,D demonstrate that AG-670 and AO-022 are potent to SCC-4 cells with IC_50_ values of 41.8 µM (95% CI: 33.5–65.3) and 154.9 µM (95% CI: 108.5–268.4), respectively.

**Figure 4:**
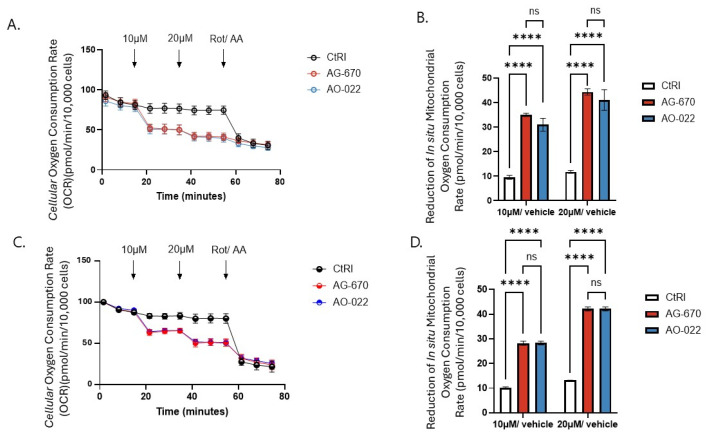
Effect of sublethal doses of NNMT inhibitors (AG-670 and AO-022) on oxygen consumption rates (OCRs) in SCC-4 and DOK cells. Cellular and reduced in situ mitochondrial oxygen consumption (OCR) in (**A**,**B**) SCC-4 and (**C**,**D**) DOK cells was determined by Seahorse XF analyzer (Agilent) in the absence and presence of 10 µM AG-670 and AO-022. The control was addition of vehicle (DMSO) alone (CtRl). Antimycin A (AntiA) and rotenone (Rot) were ultimately added to inhibit in situ mitochondrial oxygen consumption in order to quantify the cellular oxygen due to mitochondrial activity. Experiments were performed at least three time in triplicate. Data are expressed as mean + sem (*n* = 3). Key: **** *p* < 0.0001; ns, no significant difference.

We next determined the effect of sublethal doses of NNMT inhibitors (AG-670 and AO-022) on oxygen consumption rates (OCR) in SCC-4 and DOK cells (Figure 4), which are cells that have been shown to express NNMT (Figure 2). Cellular oxygen consumption rates are regularly used as an index of general cellular metabolism in primary cells and in many, but not all, cancer cells. We could demonstrate that both AG-670 and AO-022, at a sublethal dose of 10 µM, inhibit cellular oxygen consumption (Figure 4A,C) by inhibiting in situ mitochondrial oxygen consumption (~3–4 fold) in SCC-4 and DOK cells (Figure 4B,D), respectively. Similar results were observed for Hs578T cells (Figure S4), which also express NNMT (Figure 2).

**Figure 5:**
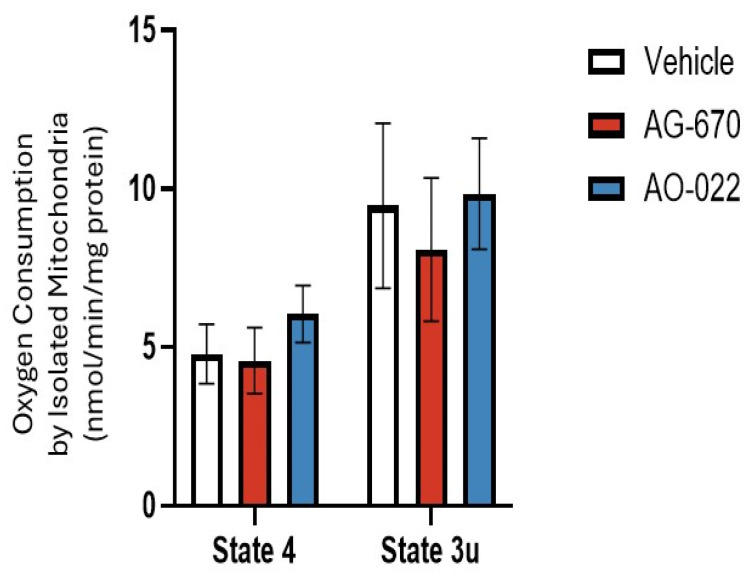
No effect of NNMT inhibitors (AG-670 and AO-022) on isolated mitochondrial activity. Oxygen consumption by isolated mitochondria from rat liver using NADH-dependent substrates (glutamate and malate) in state 4 and state 3 uncoupled rate (an index of maximal electron transport chain activity) conditions, which were measured the presence and absence of 10 µM NNMT inhibitors AG-670 and AO-022. Experiments were performed at least three time in triplicate. Data are expressed as mean + sem (*n* = 3).

As the NNMT inhibitors were designed for the nicotinamide site in NNMT, it was decided to investigate the efficacy of AG-670 and AO-022 on oxygen consumption by isolated mitochondria (Figure 5). It was demonstrated that neither inhibitor affected oxygen consumption in isolated mitochondria respiring on glutamate and malate, which are a source of matrix NADH_2_ for complex 1-initiated electron transport chain activity.

**Figure 6:**
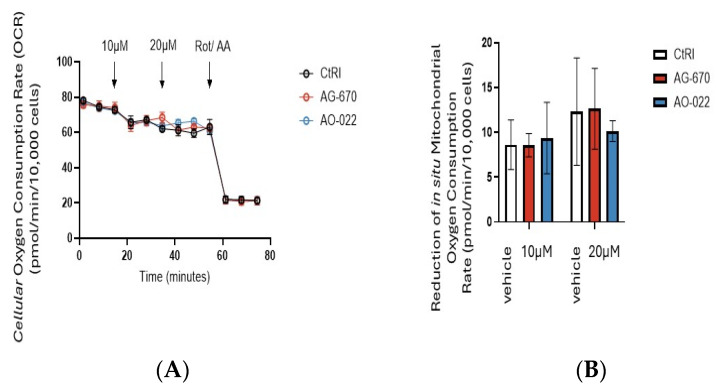
Effect of sublethal doses of NNMT inhibitors (AG-670 and AO-022) on oxygen consumption rates (OCRs) in MCF 7 cells. (**A**) Cellular and (**B**) reduced in situ mitochondrial oxygen consumption (OCR) in MCF 7 cells was determined by Seahorse XF analyzer (Agilent) in the absence and presence of 10 µM AG-670 and AO-022. The control was the addition of vehicle (DMSO) alone (CtRl). Antimycin A (AntiA) and rotenone (Rot) were ultimately added to inhibit in situ mitochondrial oxygen consumption in order to quantify the cellular oxygen due to mitochondria. Experiments were performed at least three times in triplicate. Data are expressed as mean + sem (*n* = 3).

We then determined the effect of sublethal doses of NNMT inhibitors (AG-670 and AO-022) on oxygen consumption rates (OCR) in MCF 7 cells (Figure 6), which are cells that have been demonstrated not to express NNMT (Figure 2). We demonstrated that neither AG-670 nor AO-022, at a sublethal dose of 10 µM, inhibited cellular oxygen consumption (Figure 6A) or in situ mitochondrial oxygen consumption in MCF 7 cells (Figure 6B).

## 3. Discussion

Oral squamous cell carcinoma (OSCC) accounts for up to 90% of all oral malignancies and is the sixth most common cancer worldwide. Our research was interested in finding molecular targets to treat this cancer. The enzyme, *N*-nicotinamide methyltransferase (NNMT), is one such candidate and has been shown to be overexpressed in a variety of human cancers [3,20,21,22,23,24,25,26,27]. Hence, NNMT has been investigated by several laboratories as a potential anti-cancer target [4,5,6,7,8,9,10,11]. In this paper, NNMT is shown to be expressed in oral squamous cell carcinoma (SCC-4) cells, dysplastic oral keratinocyte (DOK) cells, primary gingival keratinocyte (PGK) cells and the triple negative breast cancer cell line Hs578T. These data are consistent with NNMT expression in OSCC biopsies [3]. Furthermore, we confirm that NNMT is not expressed in MCF 7 breast cancer cells, as has been demonstrated by others [29,30]. In addition, the NNMT inhibitors selected from our pharmacophore design (Supplementary Section S1) have been demonstrated to be direct inhibitors of the enzyme NNMT and cytotoxic to SCC-4 cells. In an endeavor to examine the mechanism behind that potency, AG-670 and AO-022 were tested for efficacy on cellular oxygen consumption, which is an index of the overall metabolism in cells. It was demonstrated that sublethal doses of AG-670 and AO-022 inhibit in situ mitochondrial oxygen consumption in intact SCC-4 (and DOK and Hs578T cells), from which we deduce that a restriction of ATP supply is a prelude to, and probable factor in, the potency of the drugs. Furthermore, although the drugs were designed to target NNMT, it was important to establish whether the inhibition of in situ mitochondrial oxygen consumption in SCC-4 cells might be due to a direct effect on mitochondrial function independent of NNMT. This was addressed firstly by demonstrating that AG-670 and AO-022 do not directly inhibit the mitochondrial electron transport chain function in isolated mitochondria, and secondly, by demonstrating that AG-670 and AO-022 had no effect on in situ mitochondrial oxygen consumption in MCF 7 cells, i.e., cells that do not express NNMT. We therefore conclude that NNMT activity directly affects mitochondrial activity in situ and that the inhibitors of NNMT inhibit in situ mitochondrial oxygen consumption in SCC-4 indirectly via the inhibition of NNMT. The instant impact of the NNMT inhibitors on mitochondrial function would indicate that a decline energy metabolism is the primary event in the cytotoxic action of these drugs and hardwires NNMT activity to mitochondrial function. Possible explanations as to the mechanism behind the indirect inhibition of mitochondrial function via NNMT stem from a couple of interesting observations in the literature. Parsons et al. [31] revealed that NNMT expression contributes to cell survival by enhancing complex I activity, which is a process that appears to be mediated via the protection of the mitochondrial complex 1 subunit, NDUFS3, from degradation. The same group also demonstrated that these effects arise due to the increased production of MNA as well as demonstrating that NNMT and 1-methylnicotinamide are cytoprotective against Complex I inhibitors MPP^+^ and rotenone, which is mediated via the maintenance of Complex I activity arising from the protection of NDUFS3 from inhibitor-mediated damage. In the SH-SY5Y cell line, the expression of NNMT substantially reduced cell death, which correlated with an increase in the ATP/ADP ratio and Complex I activity. Interestingly, Liu et al. [32] demonstrated that NNMT increases mitochondrial complex I activity in situ in SH-SY5Y cells via sirtuin 3, which is a mitochondrial NAD^+^-dependent deacetylase [33,34]. Further lines of inquiry would investigate whether our inhibitors affect energy-dependent enzymes, such as AMPK and sirtuin 1 activity, and cancer phenotypes such as epithelial–mesenchymal transition, all of which are affected by NNMT activity [35,36,37]. Also, the validation of cytotoxicity across additional OSCC cell lines (e.g., SCC-9, SCC-15, SCC-25, Cal-27) would be an important next step. Future work might also seek to generate a NNMT knock-out OSCC cell line, generated by CRISPR-cas9, for instance, as a more relevant negative control to MCF-7 cells. The relative expression levels of NNMT in primary versus OSCC cells would also be worth investigating in the context of inhibitor potency.

## 4. Materials and Methods

### 4.1. In Silico Modeling

The two small molecule inhibitors of NNMT (AG-670 and AO-022) (Figure 1B,C) were purchased from Specs Compound Handling B.V./eMolecules (Zoetermeer, The Netherlands) based on our work defining the pharmacophore of the in silico nicotinamide binding site (Supplementary Information S1).

### 4.2. Cell Culture

All cells were purchased from the American Type Culture Collection (ATCC) (Manassas, VA USA) and cultured as per their recommendations. SCC-4 cells were originally derived from stage 3 tongue cancer in a 50-year-old male [38]. Dysplastic oral keratinocyte (DOK) cells were originally from the dorsal part of the tongue of a 57-year-old man [39]. MCF-7 cells are a human breast cancer cell line derived from a 69-year-old female with metastatic adenocarcinoma. It is a key luminal A subtype cell line, characterized by being estrogen (ER), progesterone (PR), and glucocorticoid receptor-positive [40]. Hs578T is a triple negative (no estrogen, progesterone and HER2 receptors) breast cancer cell line originally from epithelial cells isolated from breast tissue derived from a 74-year-old female breast cancer patient [41,42]. A small number of primary gingival keratinocyte (PGK) cells were also cultured for immunoblotting. All cells were cultured as described in Karavyraki and Porter [43].

### 4.3. Immunoblotting

The detection of NNMT was assessed by immunoblot using Novus Biologicals (Bio-Techne, Minneapolis, MN, USA) antibody NBP2-00537 with cell lysates, using the methodology described in Ge et al. [44].

### 4.4. Cytotoxicity (IC_50_)

Cell viability was determined by Alamar blue assay using a range of NNMT inhibitor concentrations over 72 h using 1% FBS.

### 4.5. Enzyme Activity (EC_50_)

An NNMT inhibitor screening assay kit (Sigma-Aldrich (Saint Louis, MO, USA), MAK229) was used to determine the potency of inhibitors on the isolated enzyme. This kit utilizes SAM as the methyl group donor and nicotinamide as the substrate. NNMT methylates nicotinamide generating S-adenosylhomocysteine (SAH) and 1-methylnicotinamide. The SAH is hydrolyzed by SAH hydrolase to form homocysteine, the free thiol group of which is detected using a thiol-detecting probe, generating an enhanced fluorescence signal (λex = 392 nm/λem = 482 nm).

### 4.6. Oxygen Consumption Rates (OCR)

Cellular and in situ mitochondrial oxygen consumption (OCR) were determined by a Seahorse XF analyzer (Agilent, Santa Clara, CA, USA) as described in Karavyraki and Porter [43]. AG-670 and AO-022 were dissolved in DMSO (vehicle). Antimycin A (AntiA) and rotenone (Rot) were ultimately added to inhibit in situ mitochondrial oxygen consumption in order to quantify the cellular oxygen due to mitochondrial activity. Oxygen consumption by isolated mitochondria from rat liver using NADH-dependent substrates (glutamate and malate) in the presence and absence of NNMT inhibitors was determined using a Rank Oxygen Electrode as described in Martin et al. [45].

### 4.7. Statistical Analysis

All data are presented as the mean ± SEM from n independent experiments (n-values specified for each figure in the figure legends). Comparisons between two groups were performed using unpaired two-tailed Student’s *t*-tests. Comparisons between three or more groups (*n*) were performed using one-way ANOVA followed by Dunnett’s or Tukey’s post hoc test for multiple comparisons as appropriate. Statistical significance was set at *p* < 0.05. IC_50_ and EC_50_ values were determined by nonlinear regression analysis using a four-parameter logistic (variable slope) model: Y = Bottom + (Top − Bottom)/(1 + 10^((LogEC_50_ − X) × HillSlope)) with 95% confidence intervals derived from the fitted curves. All statistical analyses and curve fitting were performed using GraphPad Prism (Graph Software LLC, Boston, MA, USA) (version 10.6.1).

## 5. Conclusions

This study has identified two novel small molecule inhibitors of NNMT and demonstrated that NNMT is a potential drug target for oral cancer.

## Data Availability

The original contributions presented in this study are included in the article/Supplementary Material. Further inquiries can be directed to the corresponding author.

## Supplementary Materials

The following supporting information can be downloaded at: https://www.mdpi.com/article/10.3390/ph19030516/s1, Figure S1: Ribbon diagram of NNMT-SAH-NAM ternary complex, using PDB entry: 3ROD and MOE 2020; Figure S2: Molecular Docking; Figure S3: Pharmacophore models generated using MOE, along with the feature legend. (A) Pharmacophore model for the first set of inhibitors with linker structures; (B) Pharmacophore model for the second set of cyclic inhibitors; Figure S4: Effect of potential NNMT inhibitors on OCR in Triple Negative Breast Cancer Hs578T cells; Table S1: Five compounds selected from the pharmacophore. References [46,47,48,49] are cited in the Supplementary.

## References

[1] Moore S.R., Johnson N.W., Pierce A.M., Wilson D.F. (2000). The epidemiology of tongue cancer: A review of global incidence. Oral Dis..

[2] Dong Y., Zhao Q., Ma X., Ma G., Liu C., Chen Z., Yu L., Liu X., Zhang Y., Shao S. (2015). Establishment of a new OSCC cell line derived from OLK and identification of malignant transformation-related proteins by differential proteomics approach. Sci. Rep..

[3] Sartini D., Santarelli A., Rossi V., Goteri G., Rubini C., Ciavarella D., Muzio L.L., Emanuelli M. (2007). Nicotinamide N-methyltransferase upregulation inversely correlates with lymph node metastasis in oral squamous cell carcinoma. Mol. Med..

[4] Horning B.D., Suciu R.M., Ghadiri D.A., Ulanovskaya O.A., Matthews M.L., Lum K.M., Backus K.M., Brown S.J., Rosen H., Cravatt B.F. (2016). Chemical Proteomic Profiling of Human Methyltransferases. J. Am. Chem. Soc..

[5] van Haren M.J., Zhang Y., Buijs N., Thijssen V., Sartini D., Emanuelli M., Jongkees S., Martin N. (2020). Macrocyclic Peptides as Allosteric Inhibitors of Nicotinamide N-Methyltransferase (NNMT). ChemRxiv.

[6] Neelakantan H., Vance V., Wetzel M.D., Wang H.-Y.L., McHardy S.F., Finnerty C.C., Hommel J.D., Watowich S.J. (2018). Selective and membrane-permeable small molecule inhibitors of nicotinamide N-methyltransferase reverse high fat diet-induced obesity in mice. Biochem. Pharmacol..

[7] Kannt A., Rajagopal S., Kadnur S.V., Suresh J., Bhamidipati R.K., Swaminathan S., Hallur M.S., Kristam R., Elvert R., Czech J. (2018). A small molecule inhibitor of Nicotinamide N-methyltransferase for the treatment of metabolic disorders. Sci. Rep..

[8] Ruf S., Hallur M.S., Anchan N.K., Swamy I.N., Murugesan K.R., Sarkar S., Narasimhulu L.K., Putta V.R.K., Shaik S., Chandrasekar D.V. (2018). Novel nicotinamide analog as inhibitor of nicotinamide N-methyltransferase. Bioorganic Med. Chem. Lett..

[9] Lee H.-Y., Suciu R.M., Horning B.D., Vinogradova E.V., Ulanovskaya O.A., Cravatt B.F. (2018). Covalent inhibitors of nicotinamide N-methyltransferase (NNMT) provide evidence for target engagement challenges in situ. Bioorganic Med. Chem. Lett..

[10] Babault N., Allali-Hassani A., Li F., Fan J., Yue A., Ju K., Liu F., Vedadi M., Liu J., Jin J. (2018). Discovery of Bisubstrate Inhibitors of Nicotinamide N-Methyltransferase (NNMT). J. Med. Chem..

[11] Policarpoa R.L., Decultota L., Mayb E., Kuzmičc P., Carlsonb S., Huanga D., Chua V., Wright B.A., Dhakshinamoorthy S., Kannte A. (2019). High-Affinity Alkynyl Bisubstrate Inhibitors of Nicotinamide N-Methyltransferase (NNMT). J. Med. Chem..

[12] Bromberg A., Levine J., Belmaker R.H., Agam G. (2010). Hyperhomocysteinemia does not affect global DNA methylation and nicotinamide N-methyltransferase expression in mice. J. Psychopharmacol..

[13] Kim H.C., Mofarrahi M., Vassilakopoulos T., Maltais F., Sigala I., Debigare R., Bellenis I., Hissain S.N.A. (2010). Expression and functional significance of nicotinamide N-methyl transferase in skeletal muscles of patients with chronic obstructive pulmonary disease. Am. J. Respir. Crit. Care Med..

[14] Makarov M.V., Trammell S.A.J., Migaud M.E. (2019). The chemistry of the vitamin B3 metabolome. Biochem. Soc. Trans..

[15] Xie N., Zhang L., Gao W., Huang C., Huber P.E., Zhou X., Li C., Shen G., Zou B. (2020). NAD+ metabolism: Pathophysiologic mechanisms and therapeutic potential. Signal Transduct. Target. Ther..

[16] Komatsu M., Kanda T., Urai H., Kurokochi A., Kitahama R., Shigaki S., Ono T., Yukioka H., Hasegawa K., Tokuyama H. (2018). NNMT activation can contribute to the development of fatty liver disease by modulating the NAD^+^ metabolism. Sci. Rep..

[17] Aksoy S., Szumlanski C.L., Weinshilboum R.M. (1994). Human liver nicotinamide N-methyltransferase. cDNA cloning, expression, and biochemical characterization. J. Biol. Chem..

[18] Pissios P. (2017). Nicotinamide N-Methyltransferase: More Than a Vitamin B3 Clearance Enzyme. Trends Endocrinol. Metab..

[19] Thomas M.G., Saldanha M., Mistry R.J., Dexter D.T., Ramsden D.B., Parsons R.B. (2013). Nicotinamide N-methyltransferase expression in SH-SY5Y neuroblastoma and N27 mesencephalic neurones induces changes in cell morphology via ephrin-B2 and Akt signalling. Cell Death Dis..

[20] Xu J., Moatamed F., Caldwell J.S., Walker J.R., Kraiem Z., Taki K., Brent G.A., Hershman J.M. (2003). Enhanced expression of nicotinamide N-methyltransferase in human papillary thyroid carcinoma cells. J. Clin. Endocrinol. Metab..

[21] Tomida M., Mikami I., Takeuchi S., Nishimura H., Akiyama H. (2009). Serum levels of nicotinamide N-methyltransferase in patients with lung cancer. J. Cancer Res. Clin. Oncol..

[22] Peng H., Yang H.-W., Song L.-W., Zhou Z. (2009). Screening the differential expression of adriamycin-resistance related genes of breast cancer by cDNA microarray. Zhonghua Yi Xue Za Zhi.

[23] Lim B.-H., Cho B.-I., Kim Y.N., Kim J.W., Soon-Tae P., Chang-Won L. (2006). Overexpression of nicotinamide N-methyltransferase in gastric cancer tissues and its potential post-translational modification. Exp. Mol. Med..

[24] Rogers C., Fukushima N., Sato N., Shi C., Prasad C., Hustinx S.R., Matsubayashi H., Canto M., Eshleman J.R., Hruban R.H. (2006). Differentiating pancreatic lesions by microarray and QPCR analysis of pancreatic juice RNAs. Cancer Biol. Ther..

[25] Roessler M., Rollinger W., Palme S., Hagmann M.-L., Berndt P., Engel A.M., Schneidinger B., Pfeffer M., Andres H., Karl J. (2005). Identification of nicotinamide N-methyltransferase as a novel serum tumor marker for colorectal cancer. Clin. Cancer Res..

[26] Sartini D., Muzzonigro G., Milanese G., Pierella F., Rossi V., Emanuelli M. (2006). Identification of nicotinamide N-methyltransferase as a novel tumor marker for renal clear cell carcinoma. J. Urol..

[27] Tsuchiya A., Sakamoto M., Yasuda J., Chuma M., Ohta T., Ohki M., Yasugi T., Taketani Y., Hirohashi S. (2003). Expression profiling in ovarian clear cell carcinoma: Identification of hepatocyte nuclear factor-1 beta as a molecular marker and a possible molecular target for therapy of ovarian clear cell carcinoma. Am. J. Pathol..

[28] Lu X.M., Long H. (2018). Nicotinamide N-methyltransferase as a potential marker for cancer. Neoplasma.

[29] Zhang J., Wang Y., Li G., Yu H., Xie X. (2014). Down-Regulation of Nicotinamide N-methyltransferasInduces Apoptosis in Human Breast Cancer Cells via the Mitochondria-Mediated Pathway. PLoS ONE.

[30] Yu H., Zhou X., Wang Y., Huang X., Yang J., Li G., Xie X., Zhang J. (2020). Nicotinamide N-methyltransferase inhibits autophagy induced by oxidative stress through suppressing the AMPK pathway in breast cancer cells. Cancer Cell Int..

[31] Parsons R.B., Aravinda S., Kadampeswaran A., Evans E.A., Sandhu K.K., Levy E.R., Thomas M.G., Austen B.M., Ramsden D.B. (2011). The expression of nicotinamide N-methyltransferase increases ATP synthesis and protects SH-SY5Y neuroblastoma cells against the toxicity of complex I inhibitors. Biochem. J..

[32] Liu K.Y., Mistry R.J., Aguirre C.A., Fasouli E.S., Thomas M.G., Klamt F., Ramsden D.B., Parsons R.B. (2015). Nicotinamide N-methyltransferase increases complex I activity in SH-SY5Y cells via sirtuin 3. Biochem. Biophys. Res. Commun..

[33] Nogueiras R., Habegger K.M., Chaudhary N., Finan B., Banks A.S., Dietrich M.O., Horvath T.L., Sinclair D.A., Pfluger P.T., Tschöp M.H. (2012). Sirtuin 1 and sirtuin 3: Physiological modulators of metabolism. Physiol. Rev..

[34] Zhang J., Xiang H., Liu J., Chen Y., He R.-R., Liu B. (2020). Mitochondrial Sirtuin 3: New emerging biological function and therapeutic target. Theranostics.

[35] Shin J.H., Park C.W., Yoon G., Hong S.M., Choi K.Y. (2018). NNMT depletion contributes to liver cancer cell survival by enhancing autophagy under nutrient starvation. Oncogenesis.

[36] Huang Q., Chen H., Yin D., Wang J., Wang S., Yang F., Li J., Mu T., Li J., Zhao J. (2024). Multi-omics analysis reveals NNMT as a master metabolic regulator of metastasis in esophageal squamous cell carcinoma. NPJ Precision Oncol..

[37] Hong S., Moreno-Navarrete J.M., Wei X., Kikukawa Y., Tzameli I., Prasad D., Lee Y., Asara J.M., Fernandez-Real J.M., Maratos-Flier E. (2015). Nicotinamide N-methyltransferase regulates hepatic nutrient metabolism through Sirt1 protein stabilization. Nat. Med..

[38] Rheinwald J.G., Beckett M.A. (1981). Tumorigenic keratinocyte lines requiring anchorage and fibroblast support cultures from human squamous cell carcinomas. Cancer Res..

[39] Chang S.E., Foster S., Betts D., Marnock W.E. (1992). DOK, a cell line established from human dysplastic oral mucosa, shows a partially transformed non-malignant phenotype. Int. J. Cancer.

[40] Levenson A.S., Jordan V.C. (1997). MCF-7: The First Hormone-responsive Breast Cancer Cell Line. Cancer Res..

[41] Hackett A.J., Smith H.S., Springer E.L., Owens R.B., Nelson-Rees W.A., Riggs J.L., Gardner M.B. (1977). Two syngeneic cell lines from human breast tissue: The aneuploid mammary epithelial (Hs578T) and the diploid myoepithelial (Hs578Bst) cell lines. J. Natl. Cancer Inst..

[42] Dawson S.J., Provenzano E., Caldas C. (2009). Triple negative breast cancers: Clinical and prognostic implications. Eur. J. Cancer.

[43] Karavyraki M., Porter R.K. (2022). Evidence of a role for interleukin-6 in anoikis resistance in oral squamous cell carcinoma. Med. Oncol..

[44] Ge Z., Wallace M., Turner R., Yin M., Rooney M., Porter R.K. (2025). Metabolic interplay between exogenous cystine and glutamine dependence in triple-negative breast cancer. Cell Death Discov..

[45] Martin D.S., Leonard S., Devine R., Redondo C., Kinsella G.K., Breen C.J., McEneaney V., Rooney M.F., Munsey T.S., Porter R.K. (2016). Novel mitochondrial complex I inhibitors restore glucose-handling abilities of high-fat fed mice. J. Mol. Endocrinol..

[46] Martin J.L., McMillan F.M. (2002). SAM (dependent) I AM: The S-adenosylmethionine-dependent methyltransferase fold. Curr. Opin. Struc. Biol..

[47] Peng Y., Sartini D., Pozzi V., Wilk D., Emanuelli M., Yee V.C. (2011). Structural basis of substrate recognition in human nicotinamide N-methyltransferase. Biochemistry.

[48] Meng X.-Y., Zhang H.-X., Mezei M., Cui M. (2011). Molecular docking: A powerful approach for structure-based drug discovery. Curr. Comput.-Aided Drug Des..

[49] Sethi A., Joshi K., Sasikala K., Alvala M., Gaitonde V., Karmakar P., Trivedi A. (2020). Molecular Docking in Modern Drug Discovery: Principles and Recent Applications. Drug Discovery and Development—New Advances.

