# Supplementary material for: Efficacious Anti-Cancer Drugs Targeting Nicotinamide N-Methyltransferase (NNMT) in Cultured Human Oral Squamous Cell Carcinoma (OSCC)
Source: Pharmaceuticals (Basel). 2026 Mar 22;19(3):516. doi: 10.3390/ph19030516 (PMC13028826; doi:10.3390/ph19030516)
Supplement: Supplementary file 1 [file pharmaceuticals-19-00516-s001.zip › pharmaceuticals-4174918-supplementary.pdf]

Supplementary Section S1

1.1 Nicotinamide *N*-Methyltransferase (NNMT) structure

The NNMT protein is made up of a single 264 amino acid polypeptide, with a molecular weight of 29.6 kDa, where it maps on chromosome 11 at 11q23. The 3-Dimensional (3D) crystal structure of NNMT is shown in Figure S1. NNMT possesses a class I SAM-dependent methyltransferase core fold, which is comprised of a seven-stranded β sheet flanked by two α helices on both sides near the N-terminus [46]. The substrate and SAM co-factor are deeply integrated within the molecule in this core fold. A reverse β-hairpin spanning lysine residue 203 to serine residue 212, along with the two α-helices, forms a "cap" that covers the active site of the enzyme [47]. There is also a flexible F27-H31 loop that adopts different conformations in the presence and absence of acceptor substrate [47].

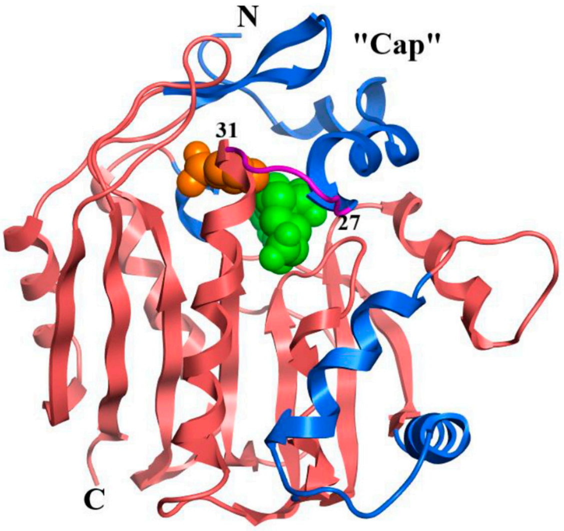

Figure S1. **Ribbon diagram of NNMT-SAH-NAM ternary complex, using PDB entry: 3ROD and MOE 2020.** The NNMT protein (chain A) is coloured red for the conserved class I SAM-dependent methyltransferase core fold, and blue for the non-conserved inserted regions characteristic of the NNMT structure, including the "Cap" over the active site and the flexible F27-H31 loop (magenta). SAH (green) and NAM (orange) are shown as space-filling spheres.

1.2 NNMT Inhibitor Database

NNMT inhibitor structures were downloaded from the protein data bank (PDB; www.rcsb.org) in sdf format. Inhibitors that were not available in the PDB were downloaded from PubChem as 2-Dimensional (2D) Structure SDF files. The PubChem files were opened in Notepad++ and saved using a file extension of ".mol" for compatibility with Molecular Operating Programme (MOE).

A database was created using MOE v2020.09 (Chemical Computing Group ULC), and each inhibitor was imported into the programme. For each inhibitor, the function "Energy Minimize" in the "Compute" tab was used to fix hydrogens and partial. This is used to find a point in configuration space where all the forces on the atoms are balanced, identifying stable conformations. Each inhibitor was added into the database in the "Database Viewer" window by selecting "Edit/New/Entry". The final database of all the existing inhibitors was subsequently compiled and created.

1.3 Molecular Docking

Guidance of molecular docking was provided by Meng et al. [48] and Sethi et al. [49]. PDB entry, 3ROD, was downloaded from the PDB and imported into MOE v2020.09. Hydrogens were added and relevant atoms were protonated at physiological pH using the "Protonate 3D" module. This determines the overall lowest potential energy configuration for the different states of terminal amides, hydroxyls, thiols, histidines, and titratable groups throughout the system. The ligand and binding pocket were isolated using "SiteView", and a molecular surface was added around the binding site to visualise the space available for the docking of ligands using the "Surface/Receptor" function. The transparency of the pocket surface was adjusted for better visualisation using the "Surface/Surfaces and Maps" panel.

Docking was carried out using the "Dock" function under the "Compute" tab. The "Receptor and Solvent Atoms" were selected in the pull-down menu beside "Receptor", and "Ligand Atoms" were selected in the "Site" pull-down menu. The inhibitor database was uploaded by setting the "Ligand" pull-down menu to "MDB File" and browsing for the existing database. The docking calculation was initiated, and all docking results were presented in a table in the "Database Viewer" window, where the poses are ranked by scores from the GBVI/WSA binding free energy calculation in the S field. The different docking poses are then compared to the ligand in the co-crystallised structure by using the "Browse" function under "File" in the database viewer, where the arrows preview each of the docked poses in the binding pocket.

1.4 Superimposition

All PFB structures were loaded into MOE and only a single copy of the protein, ligand, and surrounding solvent was retained for superimposition. All irrelevant ligands, such as glycerol, were also removed from the structure using the "SEQ" window, which opens the Sequence Editor. In addition, any issues with each structure such as ambiguous residue naming, atoms with fractional occupancies, incorrect topologies, termini capping and incorrect assignment of charges were fixed using the "Compute/Prepare/Structure Preparation" tool. All structures were subsequently protonated at physiological pH using the Protonate 3D module.

Using the Sequence Editor and the "Alignment" function, only the protein chains of each structure were selected, while the other chains were blocked. This ignores the remaining chains of each structure in sequence alignment. The "SiteFinder" tool under "Compute" was used to check different areas of the receptor where the ligands don't reach.

1.5 Pharmacophore Modelling

Using the superimposed structures, two pharmacophore queries were generated using the Pharmacophore Editor in MOE v2020.09. One pharmacophore query was created for the inhibitors with linkers, and one pharmacophore query was created for the cyclic inhibitors.

Before the features were selected, the pocket and solvent were hidden to allow a clearer view of the annotations of the superimposed ligands. In the Pharmacophore Editor, the "PCH_All" scheme was selected; a pharmacophore scheme defines the set of attributes that are used to construct ligand

annotation points which can then be matched to the query. Information on this scheme can be seen by pressing the "Show…" button in the Pharmacophore Editor, which shows the available annotation types of the scheme being used.

A series of features were created for the pharmacophores of both sets, as shown in Figure 2.4.1. The radius for each feature was adjusted using the roller wheel on the right side of "R" in the Editor to ensure it does not go outside of the structures. A total of 9 features were selected for the first set with linkers, and a total of 5 features were selected for the second set of cyclic inhibitors, where at least 6 and 4 of the highlighted features were to be present in the vendor protein screening database, respectively. Following feature selection, an exclusion volume was created around the pocket atoms. This enabled mimicking of the pocket shape, where a ligand will not match the query if any of its atom centres intersect the excluded volume. Both searches were saved to be used for the subsequent pharmacophore search using the vendor protein screening database. 17

1.6 Pharmacophore Search

The April 2021 edition of the SPECS ligand screening collection (www.specs.net) was downloaded and subsequently filtered via application of the tool, Filter (Openeye Scientific Software; www.eyesopen.com). Prior to screening, Filter uses a mix of physical property calculations and functional group knowledge to exclude undesirable compounds.

All default settings were retained and the following command was executed on a linux machine running Ubuntu 20.04.2 LTS:

Filter -dots -in Specs_SC_10mg_Apr2021.sdf -out Specs_SC_10mg_Apr2021_filter.sdf

Next, 10 conformers of all remaining compounds were generated using Omega (Openeye Scientific Software; www.eyesopen.com):

Omega -in Specs_SC_10mg_Apr2021_filter.sdf -out Specs_SC_10mg_Apr2021_filter_omega10confs.sdf -maxconfs 10.

The resulting conformer database was searched using the Pharmacophores generated previously in MOE v2020.09. The resulting database post-pharmacophore running was next docked using Fred (Openeye Scientific Software) again with default settings retained. Note that the receptor was prepared using the program 'Spruce4Docking' (Openeye Scientific Software) from the PDB entry: 6B1A). The following command was used in this case:

Fred -mpi_np 4 – receptor rec_6B1A_PROTEIN_AB__DU__6B1A.A.oedu -dbase 2021-04-22_Ph4outMatchAtLeast6+Vols.sdf -docked_molecule_file 2021-04-22_Ph4outMatchAtLeast6+Vols_FREDdocked.sdf

1.7 RESULTS

1.7.1 Final Inhibitor Database

An inhibitor database was created in MOE v2020.09 accumulating a total of 13 inhibitors.

1.7.2 Molecular Docking of Inhibitors

Molecular docking of all those13 inhibitors yielded a total of 65 entries, where 5 docking poses were generated for each inhibitor. Figure S2 demonstrates 5 different docking poses of a single compound compared to the known ligand, SAH. In general, a high negative S value is desirable, whereas RMSD is a measure of structural similarity; the closer the value to 0, the more similarity is seen between structures.

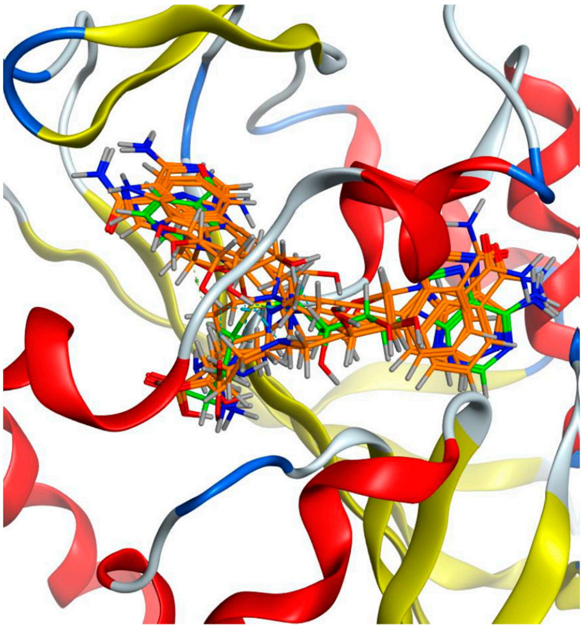

Figure S2. Molecular Docking. Molecular docking carried out in MOE, showing 5 different docking poses of LL320 (orange) docked into the binding site of NNMT, compared to SAH (green). The α-helices are coloured red, the β-sheets are coloured yellow, and the turns and ribbon loops are coloured blue and white, respectively.

1.7.3 Pharmacophore Hypothesis

Two pharmacophore models were created for two sets of compounds – one set included the inhibitors with linker structures, and the other set was composed of cyclic structure inhibitors. Figure S3 demonstrates the two final models that were used in the pharmacophore search. All top docked poses were examined visually and 5 compounds were selected for purchasing from SPECS (see Table S1) for subsequent experimental testing assessment of their activity on the Oxygen Consumption Rate (OCR) in Triple Negative Breast Cancer Hs578T cells (Fig. S4).

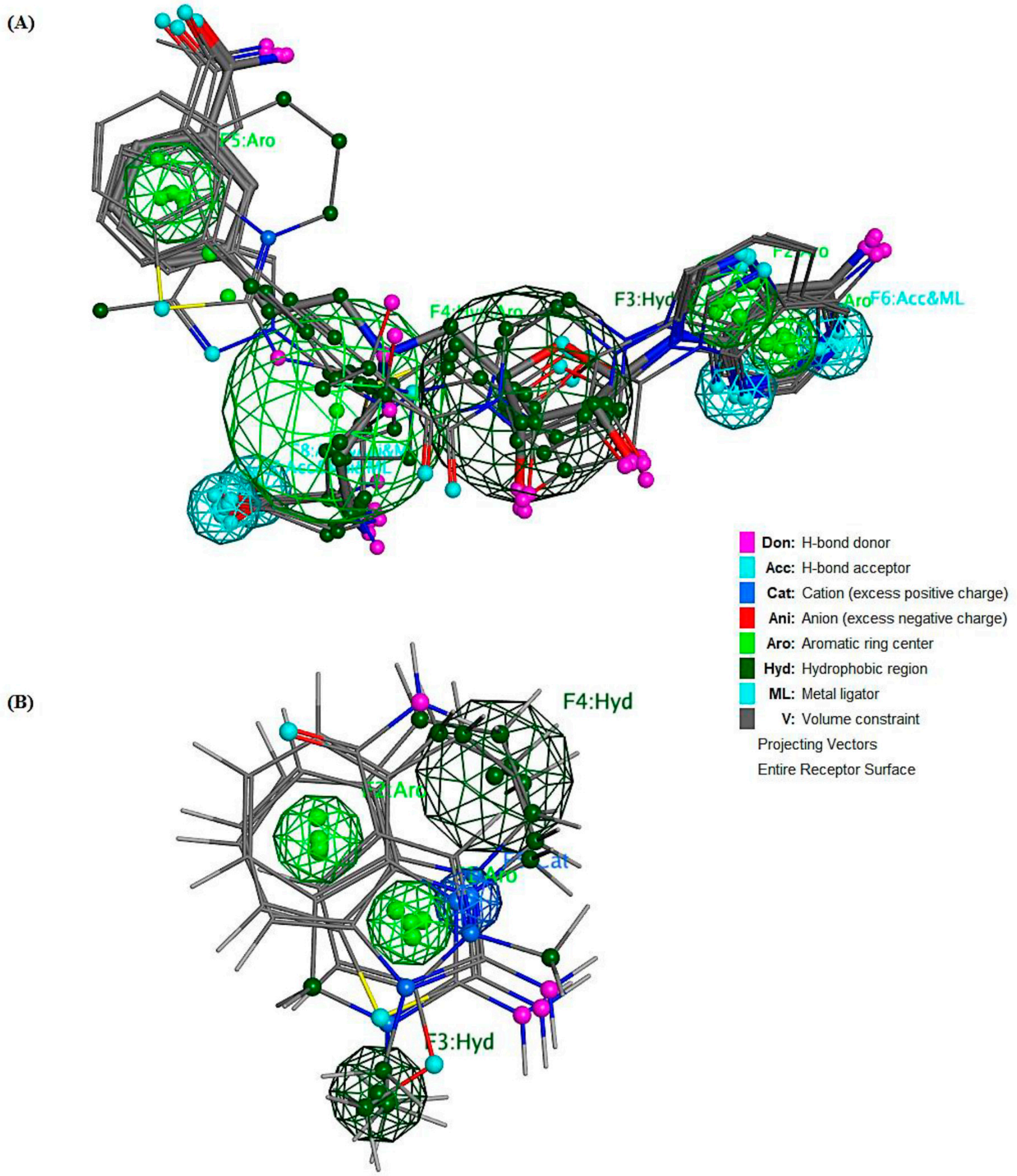

Figure S3. Pharmacophore models generated using MOE, along with the feature legend. (A) Pharmacophore model for the first set of inhibitors with linker structures; (B) Pharmacophore model for the second set of cyclic inhibitors. The first pharmacophore had the following features: F1 = Aro, F2 = Aro, F3 = Hyd, F4 = Hyd/Aro, F5 = Aro, F6 = Acc&ML, F7 = Acc&Ani&ML, F8 = Acc&Ani&ML, and F9 = Acc&ML. The second pharmacophore had the following features: F1 = Aro, F2 = Aro, F3 = Hyd, F4 = Hyd, and F5 = Cat.

Table S1. Five compounds selected from the pharmacophore

Compound	Chemical Structure	Chemical Formula	Molecular Weight
AK-968/41171495<br>(AK-968 (#2))	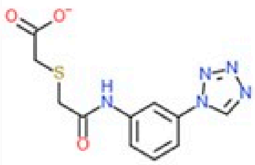	$C_{11}H_{10}N_5O_3S$	292.2934 g/mol
AQ-088/42013777<br>(AQ-088)	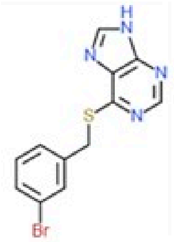	$C_{12}H_9BrN_4S$	321.1953 g/mol
AG-670/11416031<br>(AG-670)	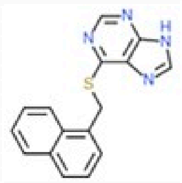	$C_{16}H_{12}N_4S$	292.3578 g/mol
AO-022/43513619<br>(AO-022)	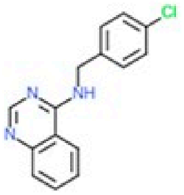	$C_{15}H_{12}ClN_3$	269.7284 g/mol
AK-968/40730313<br>(AK-968 (#1))	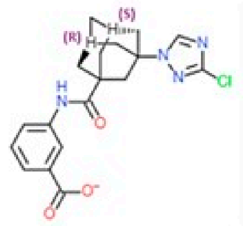	$C_{20}H_{20}ClN_4O_3$	399.85 g/mol

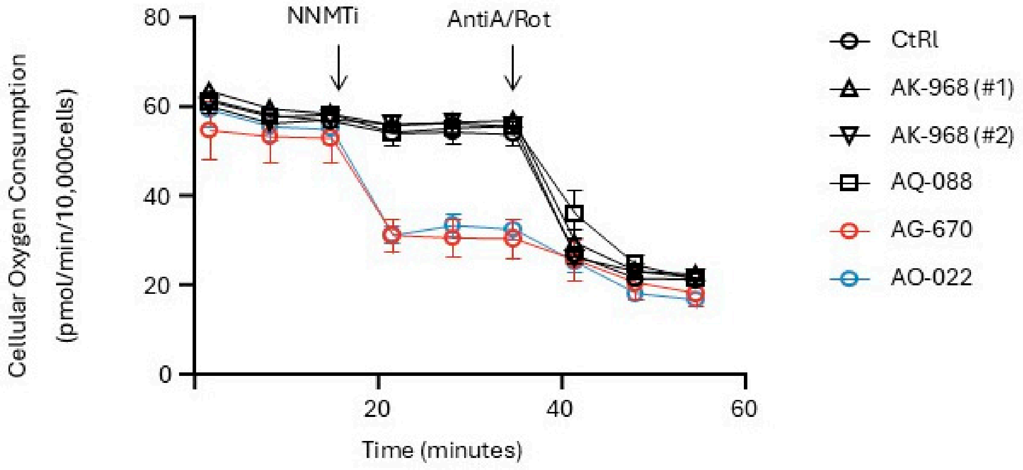

Figure S4. Effect of potential NNMT inhibitors on OCR in Triple Negative Breast Cancer Hs578T cells. Effect of acute addition of 10μM NNMT of each of the inhibitors (NNMTi) listed in Table S1 AK-968/40730313 (#1), AK-968/ 41171495(#2); AQ-088/42013777, AG-670/11416031 & AO-022/43513619) on oxygen consumption rates (OCR) in Hs578T cells. The control was addition of vehicle (DMSO) alone (CtRl). Cellular OCR was determined using a Seahorse XF analyser (Agilent). Antimycin A (AntiA) and rotenone (Rot) were ultimately added to inhibit *in situ* mitochondrial oxygen consumption in order to determine the cellular oxygen due to mitochondria. Only AG-670 & AO-022 inhibited *in situ* mitochondrial oxygen consumption.
